# NLRP3 inflammasome regulates astrocyte transformation in brain injury induced by chronic intermittent hypoxia

**DOI:** 10.1186/s12868-022-00756-2

**Published:** 2022-11-27

**Authors:** Ningning She, Yewen Shi, Yani Feng, Lina Ma, Yuqi Yuan, Yitong Zhang, Zine Cao, Xi Chen, Bingjie Zhao, Haiqin Liu, Xiaoyong Ren

**Affiliations:** Department of Otorhinolaryngology Head and Neck Surgery, The Second Affiliated Hospital of Xi’an Jiaotong University, No.157, Xiwu Road, Xi’an, 710004 Shaanxi, People’s Republic of China

**Keywords:** NLRP3, Caspase-1, ASC, IL-1β, Astrocytes, CIH Animal models

## Abstract

**Background:**

Obstructive sleep apnea (OSA) is mainly characterized by sleep fragmentation and chronic intermittent hypoxia (CIH), the latter one being associated with multiple organ injury. Recently, OSA-induced cognition dysfunction has received extensive attention from scholars. Astrocytes are essential in neurocognitive deficits via A1/A2 phenotypic changes. Nucleotide oligomerization domain (NOD)-like receptor protein 3 (NLRP3) inflammasome is considered the most important factor inducing and maintaining neuroinflammation. However, whether the NLRP3 regulates the A1/A2 transformation of astrocytes in CIH-related brain injury remains unclear.

**Methods:**

We constructed an OSA-related CIH animal model and assessed the rats' learning ability in the Morris water maze; the histopathological assessment was performed by HE and Nissl staining. The expression of GFAP (astrocyte marker), C3d (A1-type astrocyte marker), and S100a10 (A2-type astrocyte marker) were detected by immunohistochemistry and immunofluorescence. Western blotting and RT-qPCR were used to evaluate the changes of A1/A2 astrocyte-related protein and NLRP3/Caspase-1/ASC/IL-1β.

**Results:**

The learning ability of rats decreased under CIH. Further pathological examination revealed that the neurocyte in the hippocampus were damaged. The cell nuclei were fragmented and dissolved, and Nissl bodies were reduced. Immunohistochemistry showed that astrocytes were activated, and morphology and number of astrocytes changed. Immunofluorescence, Western blotting and RT-qPCR showed that the expression of C3d was increased while S100a10 was decreased. Also, the expression of the inflammasome (NLRP3/Caspase-1/ASC/IL-1β) was increased. After treatment of MCC950 (a small molecule inhibitor of NLRP3), the damage of nerve cells was alleviated, the Nissl bodies increased, the activation of astrocytes was reduced, and the expression of A2-type astrocytes was increased. In contrast, A1-type astrocytes decreased, and the expression of inflammasome NLRP3/Caspase-1/ASC/IL-1β pathway-related proteins decreased.

**Conclusion:**

The NLRP3 inflammasome could regulate the A1/A2 transformation of astrocytes in brain injury induced by CIH

**Supplementary Information:**

The online version contains supplementary material available at 10.1186/s12868-022-00756-2.

## Background

OSA is a common sleep breathing disorder. Repeated upper airway collapse and sleep obstruction can result in CIH, hypercapnia, hypoventilation, and sleep structure disturbances, which may lead to damage to multiple organ systems [1]. Injury to the central nervous system (CNS) caused by OSA has recently attracted increasing attention. Among cases of OSA, neurocognitive deficits occur at a high frequency, and these deficits can affect many cognitive domains, leading to reduced work efficiency, an increased prevalence of Alzheimer's disease, and a severe burden on families and society [2–4]. Previous scholars have used genomics, proteomics, and metabolomics to explore the common markers of OSA and neurodegenerative diseases through cerebrospinal fluid and blood, aiming to further understanding of the pathophysiological process of OSA-related neurodegenerative diseases [5–7]. It is believed that OSA-related neurocognitive deficits is caused by multiple factors such as inflammation, oxidative stress, and metabolic disorders [8]. However, the exact mechanism remains unclear and should be addressed by further research.

Astrocytes, the most numerous glial cells in the CNS, have various important roles in regulating homeostasis, increasing synaptic plasticity, providing neuroprotection, and maintaining normal brain function [9]. Under conditions of pathological activation, resting astrocytes transform into reactive astrocytes, which can be divided into A1-type neurotoxic astrocytes (marked by C3d) and A2-type protective astrocytes (represented by S100a10) [10]. A1 neurotoxic astrocytes can highly upregulate many classical complements cascade genes and induce neural damage, while A2 astrocytes, known as neuroprotective glial cells, release various neurotrophic factors that regulate brain homeostasis. Whether the phenotypic transition of A1/A2 astrocytes is involved in CIH-related neurocognitive deficits is currently unknown.

Neuroinflammation is an autoimmune response induced by CNS-related injury, such as infection, aging, or other stimulation. While moderate neuroinflammation has a protective role in CNS injury and helps maintain body homeostasis, excessive neuroinflammation can cause CNS injury. Neuroinflammation is considered important pathogenesis of various neurodegenerative diseases such as Alzheimer [11] and traumatic brain injury [12]. Inhibition of neuroinflammation is also believed to have potential therapeutic value in OSA-mediated neurocognitive deficits [13]. The NLRP3 inflammasome pathway (NLRP3/Caspase-1/ASC/IL-1β) is the core of the initiation and maintenance of neuroinflammation, mainly composed of NLRP3 oligomers, apoptosis-associated speck-like protein (ASC), and pro-caspase-1, which specifically cleave to IL-1β and IL-18 and induce inflammatory cascades [14]. In their study, Díaz-García et al*.* [15] comparatively analyzed the key components of NLRP3 inflammasome in plasma and monocytes of OSA patients. They suggested that NLRP3 activation might be an important connection mechanism between CIH and systemic inflammatory response. Wu et al*.* [16] revealed that the OSA animal model knocked out NLRP3 and exerted a central neuroprotective effect by promoting mitophagy via the PINK1-Parkin pathway. These studies demonstrated that the NLRP3 gene was a potential therapeutic target for OSA-related neurocognitive disorders. However, whether the NLRP3 inflammasome regulates A1/A2 astrocyte transformation leading to CIH-related neurocognitive deficits remains unclear.

Therefore, in this study, we analyzed the expression of A1/A2 astrocytes and NLRP3/Caspase-1/ASC/IL-1β inflammasome signaling pathway under CIH, and compared the changes of astrocytes before and after the intervention of NLRP3 inflammasome inhibitor MCC950.

## Materials and methods

### Animals and groups

Male adult Sprague–Dawley (SD) rats, weighing 160–200 g, which were provided by the Experimental Animal Center of Xi’an Jiaotong University (License no. SCXK, Shaanxi, 2012–003), were used for all the experiments. Rats were housed in groups of 4–5 per cage (545 × 395 × 200 mm), adaptively reared for 3 days, and kept under strictly controlled laboratory conditions (a temperature of 22 ± 1 °C, relative humidity of 50 ± 1%, and a light/dark cycle of 12/12 h). All protocols and procedures were approved by the Biomedical Ethics Committee of Animal Experiments of Shaanxi Province in China and complied with the principles and procedures of the Guidance Suggestions for the Care and Use of Laboratory Animals formulated by the Ministry of Science and Technology of People’s Republic of China.

The study was divided into two experimental phases. In the first stage, we evaluated the histopathologic changes in different CIH cycles, while the second stage was used for drug intervention. In the first stage, rats were divided into 4 groups: control (normoxia treatment), CIH 2 weeks (w), CIH 4w, and CIH 6w groups; in the second stage, rats were divided into two groups: control group (CIH 6w + PBS intervention), and MCC950 group (CIH 6w + MCC950 intervention). Nine rats were randomly assigned to each group. The experimental design is shown in Additional file 1: Figure S1a.

### CIH animal model

The CIH model was established according to Gozal et al. [17, 18]. Intermittent hypoxia chamber (70 × 50 × 50 cm) achieves hypoxia/reoxygenation by using an automated nitrogen/air delivery system. The O_2_ concentration was continuously measured by an O_2_ analyzer and deviations from the desired concentration were met by addition of N2 or room air through solenoid valves. Gas was circulated in the chambers at 60 L/min, in order to achieve a cyclical pattern of 10 and 21% O_2_ every 90 s. A 12-h (6:00 a.m. to 6:00 p.m.) CIH intervention was performed every day to construct a model of moderate to severe OSA. The rats in the control group were put in the same cabin while exposed to a normal environment without hypoxic intervention. During the experiment, the hypoxia of rats was observed and the body weight of rats was compared and recorded.

### Treatment of NLRP3 inflammasome inhibitor

MCC950 (AbMole BioScience, USA) is a potent and selective small molecule inhibitor of NLRP3 that can block NLRP3 activation at nanomolar concentrations. MCC950 was dissolved in DMSO as a stocked solution and then diluted with PBS as a work solution. According to the previous research [19], MCC950 was administered intraperitoneally to rats (10 mg/kg) at day 3, 4, and 5 post-modeling and then once every 2 days. The rats in the control group were intraperitoneally injected with PBS (10 mg/kg) at the same time.

### Morris water maze test

The spatial learning and memory abilities of rats under the different experimental conditions, were assessed in the Morris water maze. The water maze, which was 120 cm in diameter and 45 cm in height, filled with 25 cm of water (25 ± 1 °C) and divided into four quadrants. Also, a circular escape platform with a diameter of 12 cm was positioned 2 cm below the water surface in the III quadrant. A high-definition camera was fixed above the round barrel to record the entire swimming trajectory of the animal. The place learning experiment was conducted for 5 consecutive days, and the training was performed 4 times a day at a fixed time period, with an interval of 1 h for each experiment. In turn, the rats were put into the water from the four quadrants and searched for the underwater escape platform. Each test lasted 90 s. The experiment ended when the rats reached the underwater platform and stayed there for 3 s. If the rat did not find the platform within 90 s, it was guided to the platform and remained there for 30 s. The time to find the platform (escape latency) was collected and recorded by the video equipment, and the average value of the four training sessions of the rat was taken as the score every day. On the last day, marked the platform position in the recording system (Ethovision System, The Netherlands) and then remove the platform for a probe trial. The rats were put into the water from a fixed position, and the number of crossings of the area (12 cm in diameter circular area) corresponding to the platform original placement and the time spent on the target quadrant (the quadrant in which the platform was originally placed) were recorded within 90 s.

### Western blotting

The rats were sacrificed according to different experimental cycles, and the bilateral hippocampi were immediately removed and stored in liquid nitrogen until further processing. Fifty milligrams of hippocampal tissue were used for Western blotting analysis as previously described [20]. Experimental procedures are explained in detail in Additional file 7. β-actin was chosen as an internal control to ensure equivalent amounts of protein. Densitometric quantification of the bands was performed using Image J software (version 1.29x: NIH, Bethesda, MD, USA). The following primary antibodies were used: NLPR3 (cat. no. WL02635, 1:1000, Wanleibio, China), Caspase-1 (cat. no. sc-56036, 1:1000, Santa Cruz, USA), ASC (cat. no. sc-514414, 1:500, Santa Cruz, USA), IL-1β (cat. no. sc-12742, 1:500, Santa Cruz, USA), GFAP (cat. no. BM0055, 1:1000, Boster biological technology, China), S100a10 (cat. no. 11250–1-AP, 1:1000, Proteintech, China), C3d (cat. no. AF2655, 1:1000, RD system, USA), and β-actin (cat. no. BM3873, 1:10,000, Boster biological technology, China). The original unedited blots were presented in Additional file 2: Figure S2, Additional file 3: Figure S3, Additional file 4: Figure S4 and Additional file 5: Figure S5.

### Reverse transcription‑quantitative PCR (RT‑qPCR)

mRNA was purified by RNAiso Plus (Takara, Japan), followed by reverse transcription experiments (PrimeScript^TM^RT Master Mix; Takara, Japan) and quantitative real-time PCR (TB Green premix Ex Taq™ II; Takara, Japan). Reverse transcription was performed at 37 °C for 15 min, followed by 85˚C for 5 s and 4 °C hold. The RT‑qPCR thermocycling conditions were as follows: Initial denaturation at 95 °C for 30 s; 40 cycles of denaturation at 95 °C for 5 s, and annealing and elongation at 60 °C for 30 s. β-actin was used as the internal control. The specific gene primers that were used are shown in Additional file 6: Table S1. The gene quantification was analyzed using the 2− ^ΔΔCt^ method.

### Paraffin section preparation

Rats were anesthetized with pentobarbital sodium and transcardially perfused with 250 ml normal saline and 250 ml 4% paraformaldehyde in 0.1 mol/L phosphate buffer. Next, their brains were harvested, fixed with paraformaldehyde, embedded in paraffin. Then, a professional tissue pathologist used a rotary slicer to continuously cut (5 μm) rat brain tissue from the coronal plane. When the hippocampal area appears, continuous slices were carried out to make it adhere to the anti-unloading glass slide and bake at 62 °C.

A total of 13 sections were obtained, of which the first and last sections were stained with HE to ensure that all sections in the middle part contained the hippocampus. The second and third sections were then used for Nissl staining and HE staining. The next three sections were used for immunohistochemistry, immunofluorescence (GFAP/C3d) and immunofluorescence (GFAP/S100a10), respectively, and the slices were repeated 3 times.

### Hematoxylin eosin staining (HE)

Rats were anesthetized with sodium pentobarbital and transcardially perfused. The brains were harvested, and paraffin sections of rat brain tissues were prepared for HE staining and observed under a light microscope. Experimental procedures are explained in detail in Additional file 7. Under a 20 × optical microscope, two fields of view were randomly selected for each film in different subregions of hippocampal CA1, CA3, and DG.

### Nissl staining

Rat brain tissue sections were routinely dewaxed and hydrated, and the operation was performed according to the instructions of the Nissl Staining Solution (Nissl Staining Solution, Solarbio, China). Under a 40 × optical microscope, two fields of view were randomly selected for each film in different subregions of hippocampal CA1, CA3 and DG to observe the Nissl bodies.

### Immunohistochemical staining

Sections were routinely dewaxed and washed, after which they were stained by SP method. Antigens were retrieved with sodium citrate buffer. The remaining steps were performed according to the instructions of SP kit (ZSGB-BIO, China). Sections were incubated with mouse anti-GFAP polyclonal antibody (Cat. No. BM0055, 1:200, Bobst Biotechnology, China) overnight at 4 °C, following which Diaminobenzidine was used as a developer, and hematoxylin was used as a counterstain. Finally, they were observed under an optical microscope. Experimental procedures are explained in detail see in Additional file 7. Each specimen was randomly selected in 5–6 different fields of view according to different hippocampal regions (CA1, CA3, DG) under a 40 × microscope to count the number of GFAP + cells. Under a 40 × microscope, 5 morphologically intact astrocytes were randomly selected from each specimen for Sholl analysis. The specific steps are as follows: GFAP signal was segmented with the threshold tool and converted to binary mask before its skeletonization with the skeletonize tool. The latter tool allowed to obtain segment length and any possible bifurcation of the skeletonized image analyzed with the Fiji-Image J software. Then, the number of synaptic junctions, synaptic end-point voxels, synaptic branches and the average branch length were measured with the Sholl plugin.

### Immunofluorescence staining

Brain tissue sections were processed in the same way as immunohistochemical staining. Sections were mixed with goat polyclonal C3d antibody (cat. no. AF2655, 1:100, RD system, USA), mouse polyclonal GFAP antibody (cat. no. BM0055, 1:200, Boster biological technology, China), and rabbit polyclonal S100a10 antibody (cat. no. 11250–1-AP, 1:100, Proteintech, China), and were incubated overnight at 4 °C. After removing the sections and washing 3 times, the sections were incubated with donkey anti-mouse, donkey anti-rabbit, and donkey anti-goat secondary antibodies labeled with Alexa Fluor 488 and Alexa Fluor 647 (1:200, Jackson, UK) for 1 h at room temperature. After rinsing 3 times, the slides were mounted with an anti-fluorescence quenching mounting medium (Beyotime Biotechnology, China) containing 4’, 6-diamino-2-phenylindole (DAPI), and images were acquired using a fluorescence microscope ((Nikon Eclipse Ti-S, Japan).

### Statistical analysis

SPSS 22.0 software was used for all statistical analyses. All values for each group are presented as mean ± SD. Parametric and nonparametric tests were used according to the homogeneity of variance. According to different comparison situations, statistical differences were analyzed using Student’s t-test or one-way ANOVA, as appropriate, with Turkey’s multiple comparisons test. *P* < 0.05 was considered statistically significant.

## Results

### CIH induced the decline of spatial learning performance and the disorder of hippocampal neurons in rats

In the process, the rats in the control group were active, with shiny hair, and quick response. However, the rats in the CIH group were observed to be dispirited, unresponsive, and subject to hypoxia symptoms and signs, such as waking up as a result of laboured breathing, lifting of the head to breathe, and deepened and quickened abdominal breathing. With the prolongation of the hypoxia cycle, the rats gradually developed piloerection, curled up, and piled up, which were eventually followed by cyanosis of the lips and even lethargy. The body weight of the rats was compared, and it was found that their weight slowly increased, compared with the control group, the weight increase trend in the CIH group was slowed down (all *P* < 0.0001), as shown in Additional file 1: Figure S1b.

**Fig. 1:**
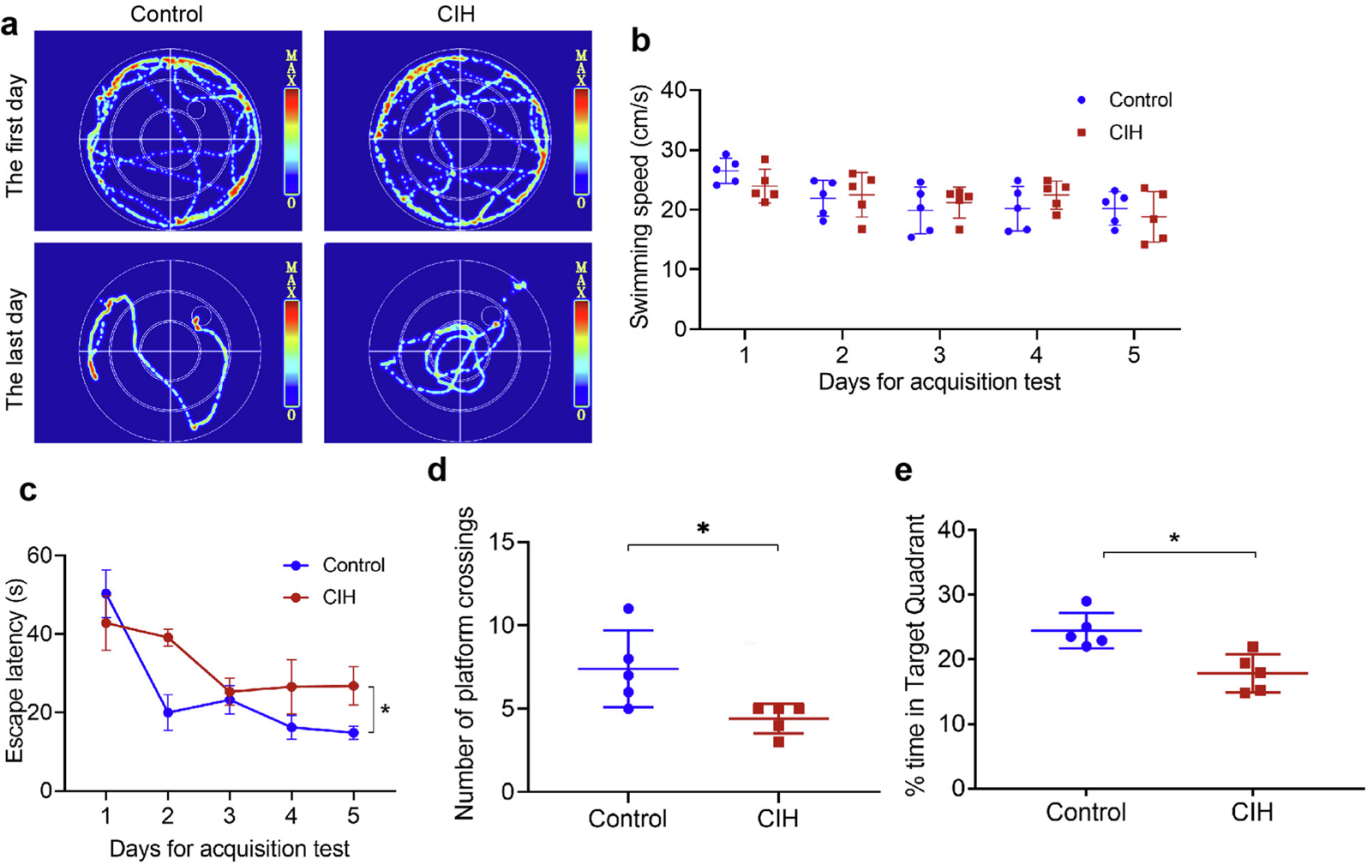
CIH induced the decline of spatial learning performance of rats. **a** The representative trajectories of the rats in the place learning experiment. **b** Comparison of swimming speed between the two groups of rats. **c** Comparison of escape latency of rats in the place learning experiment. **d** Comparison of the number of rat crossing platforms. **e** The dwell time of two groups of rats in the target quadrant. Data are presented as the mean ± SD. An unpaired t-test was used to compare the swimming speed, the number of platform crossings, and time in the target quadrant. The escape latency was analyzed using two-way ANOVA. There were 5 rats in each group for the experimental test. *P < 0.05

In the place learning experiment, Fig. 1a shows the typical swimming trajectories of rats in each group on the first and last day. Then we compared the swimming speed every day, finding no significant difference between the two groups (Fig. 1b). With the increase in the number of test days, the time required by the rats to find the platform progressively reduced (Fig. 1c). However, in comparison to the control group rats, the CIH group rats showed a longer escape latency (*P* < 0.05). In the probe trial test, the platform was removed; compared with the control group, the number of times rats crossed the platform and the time spent on target quadrant in the CIH group were significantly lower (both *P* < 0.05, Fig. 1d, e). These results suggested the CIH group performed poorly in spatial learning tasks.

**Fig. 2:**
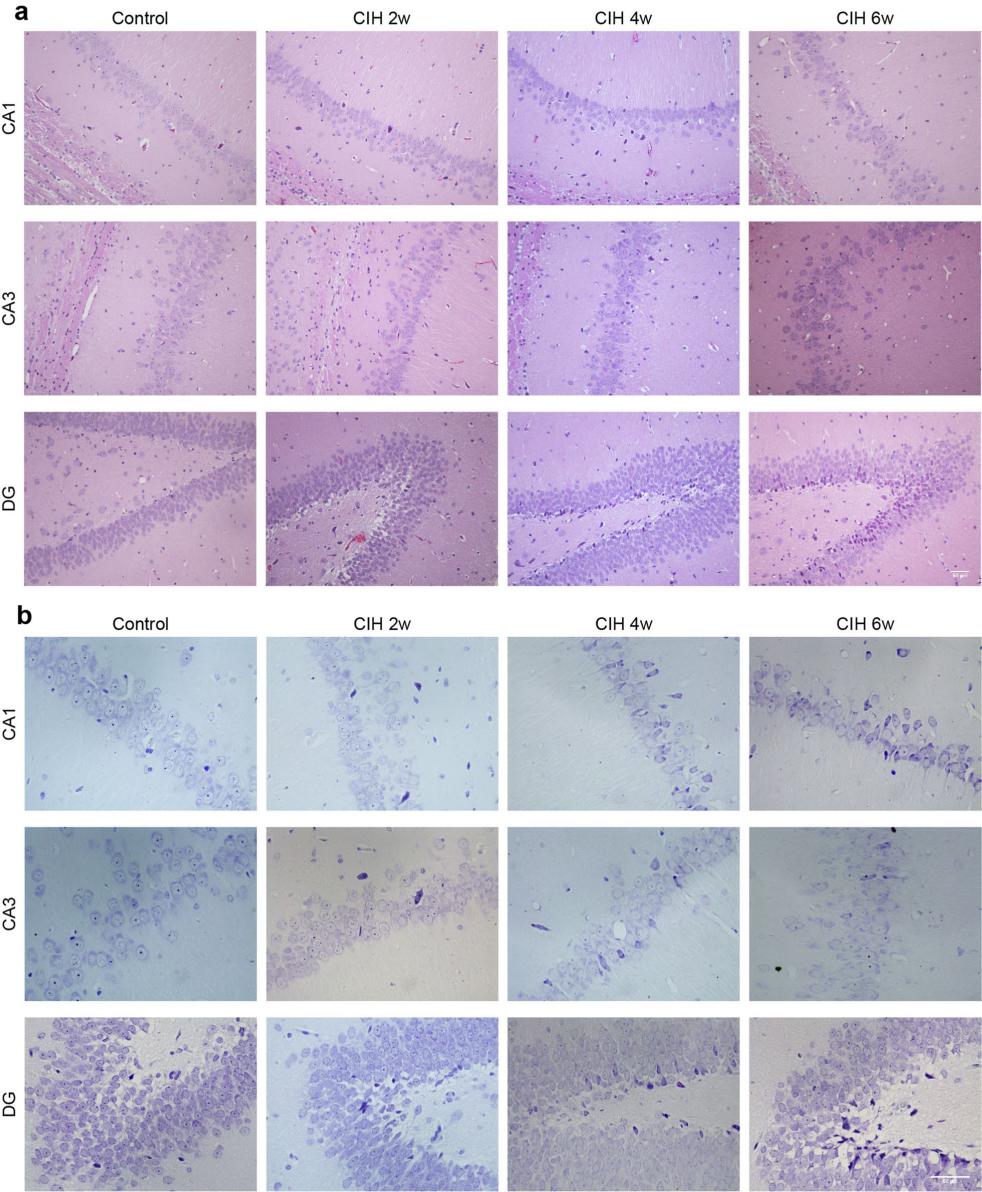
CIH induced pathological changes in rat hippocampus. **a** HE staining of different regions of the hippocampus. **b** Nissl staining of different hippocampal regions. Scale bar = 50 μm

The hippocampus is the brain tissue most sensitive to ischemia and hypoxia. HE staining showed that the control group rats had dense and orderly neurons with clear cell structure, distinct nucleoplasm in the hippocampal CA1, CA3 and DG regions. However, the CIH group showed neurons were arranged loosely and disorderly, some cells had nuclear pyknosis and fragmentation, and the damage was aggravated with the CIH cycle (Fig. 2a). Nissl staining showed that after CIH, the cellular structure of the hippocampus was damaged, Nissl bodies were fragmented, and reduced (Fig. 2b).

### CIH activates astrocytes

**Fig. 3:**
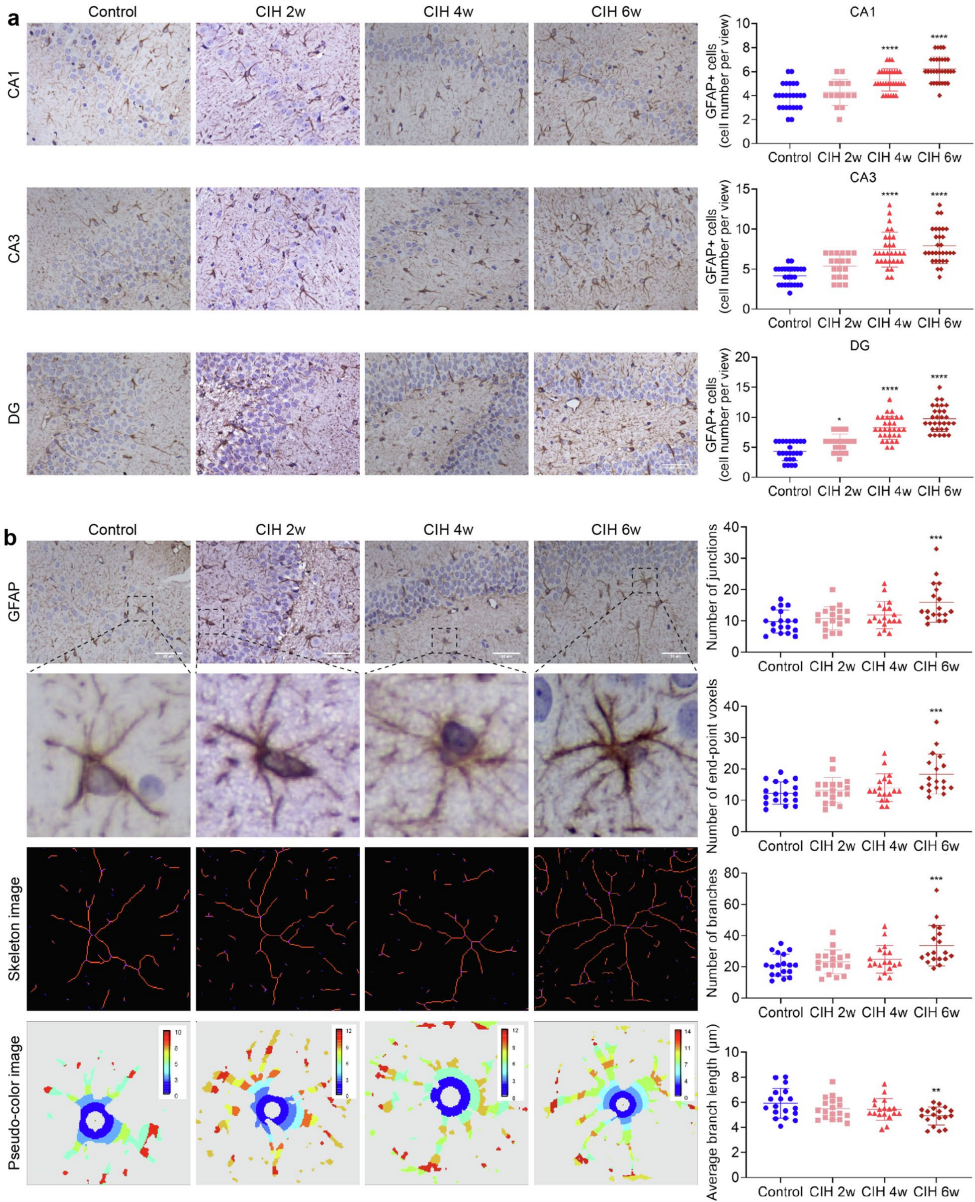
CIH induced activation of astrocytes. **a** GFAP immunohistochemical staining and GFAP + cell count in each subregion of the hippocampus. **b** Sholl analysis of morphological changes of astrocytes under CIH. Scale bar = 50 μm. Data are presented as the mean ± SD. ANOVA with Tukey’s post hoc test was used to compare the control, CIH 2w, CIH 4w, and CIH 6w group. There were three rat brain slices in each group, and 5–6 visual fields were randomly selected according to different subregions for statistical analysis. *P < 0.05, ***P < 0.001, ****P < 0.0001

Immunohistochemistry showed that astrocytes were activated under CIH (Fig. 3). After further counting the number of astrocytes in each subregion, we found that, compared with the control group, in CIH 4w group and CIH 6w group, the number of astrocytes in each subregion of the hippocampus was significantly increased (all *P* < 0.0001, Fig. 3a). However, in CIH 2w group, only astrocytes in the DG area increased significantly (*P* < 0.05, Fig. 3a), which may be related to the different sensitivity of hippocampal subregions to hypoxia.

Sholl analysis revealed that in the CIH 6w group, the number of synaptic junctions, synaptic end-point voxels, and synaptic branches were significantly increased (all *P* < 0.001, Fig. 3b), and the average branch length was significantly shortened compared with the control group (*P* < 0.01, Fig. 3b).

### CIH induces the transformation from A2 to A1 of astrocytes

**Fig. 4:**
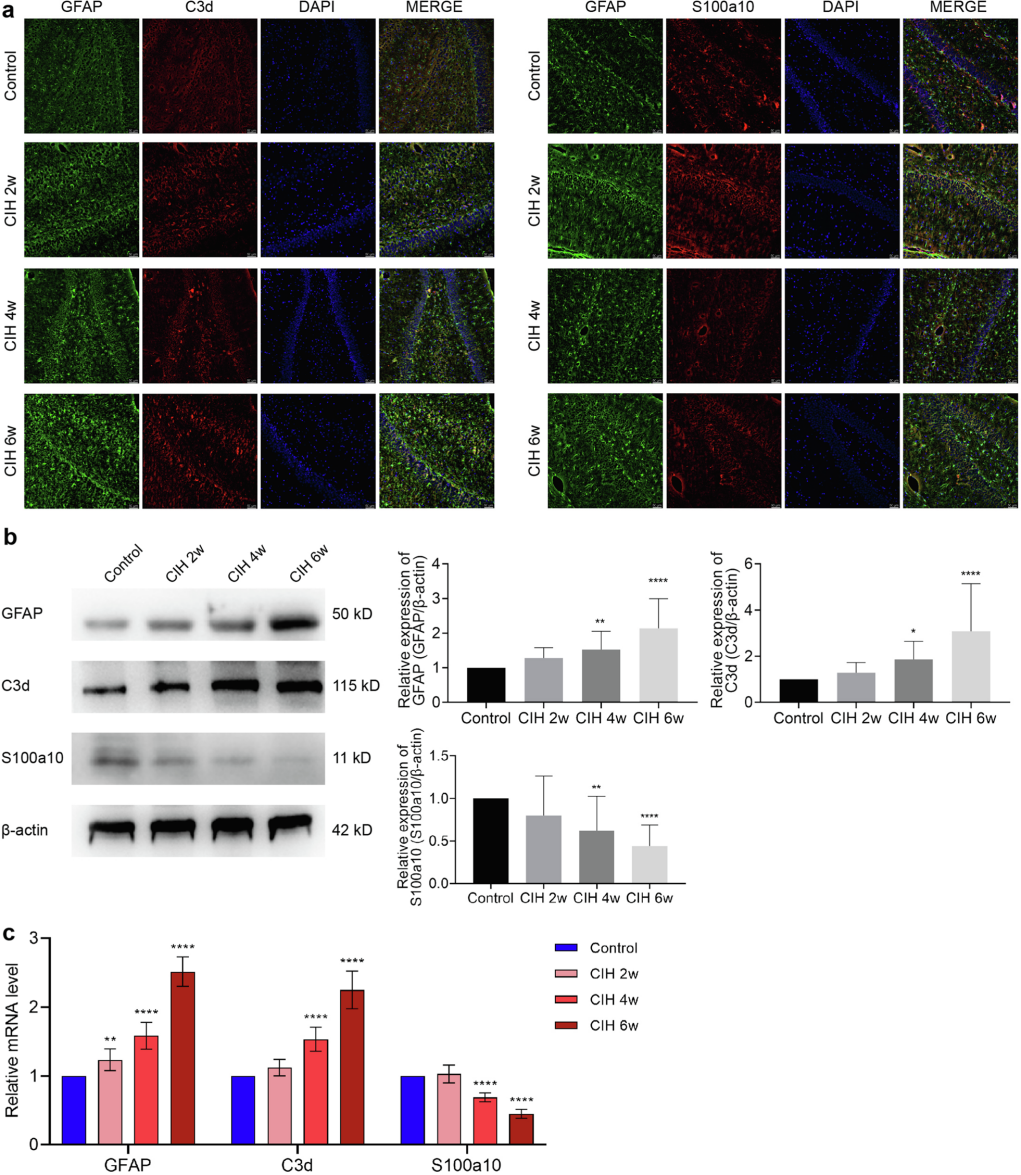
CIH induced A1/A2 phenotype astrocyte transformation, with the increase of A1 type. **a** Immunofluorescence images of astrocytes. The astrocytes were labeled by anti-GFAP (green), anti-C3d (red), and anti-S100a10 (red).** b** Expression of GFAP, C3d, and S100a10 was detected by Western blotting. **c** The mRNA expression of GFAP, C3d, and S100a10. Scale bar = 50 μm. Data are presented as the mean ± SD. ANOVA with Tukey’s post hoc test was used to compare the control, CIH 2w, CIH 4w, and CIH 6w groups. For RT-qPCR experiments, each group consisted of 4 rats, and the experiment was repeated 3 times; for western blotting, which was repeated 4 times, there were 5 rats per group. *P < 0.05, **P < 0.01, ***P < 0.001, ****P < 0.0001

Immunofluorescence double-labeling revealed that compared with the control group, the expression of S100a10 + /GFAP + positive astrocytes decreased after CIH, while the expression of C3d + /GFAP + positive astrocytes increased (Fig. 4a). Western blotting showed that compared with the control group, the expression of GFAP and C3d proteins increased with the prolongation of the CIH cycle and significantly increased at CIH 4w (*P* < 0.01; *P* < 0.05) and CIH 6w (*P* < 0.0001; *P* < 0.0001). However, the S100a10 protein decreased with the prolongation of the CIH cycle, and this change was most obvious at CIH 4w (*P* < 0.01) and 6w (*P* < 0.0001), as shown in Fig. 4b. The results of mRNA were consistent with Western blotting, as shown in Fig. 4c. Compared with the control group, the mRNA expression levels of GFAP, C3d, and S100a10 were changed in CIH 4w (all *P* < 0.0001) and CIH 6w (all *P* < 0.0001). Interestingly, the mRNA level of GFAP was also significantly elevated at CIH 2w (*P* < 0.01), which might be related to the changes in mRNA levels rather than protein levels.

### CIH activates NLRP3/Caspase-1/ASC/IL-1β signaling pathway

**Fig. 5:**
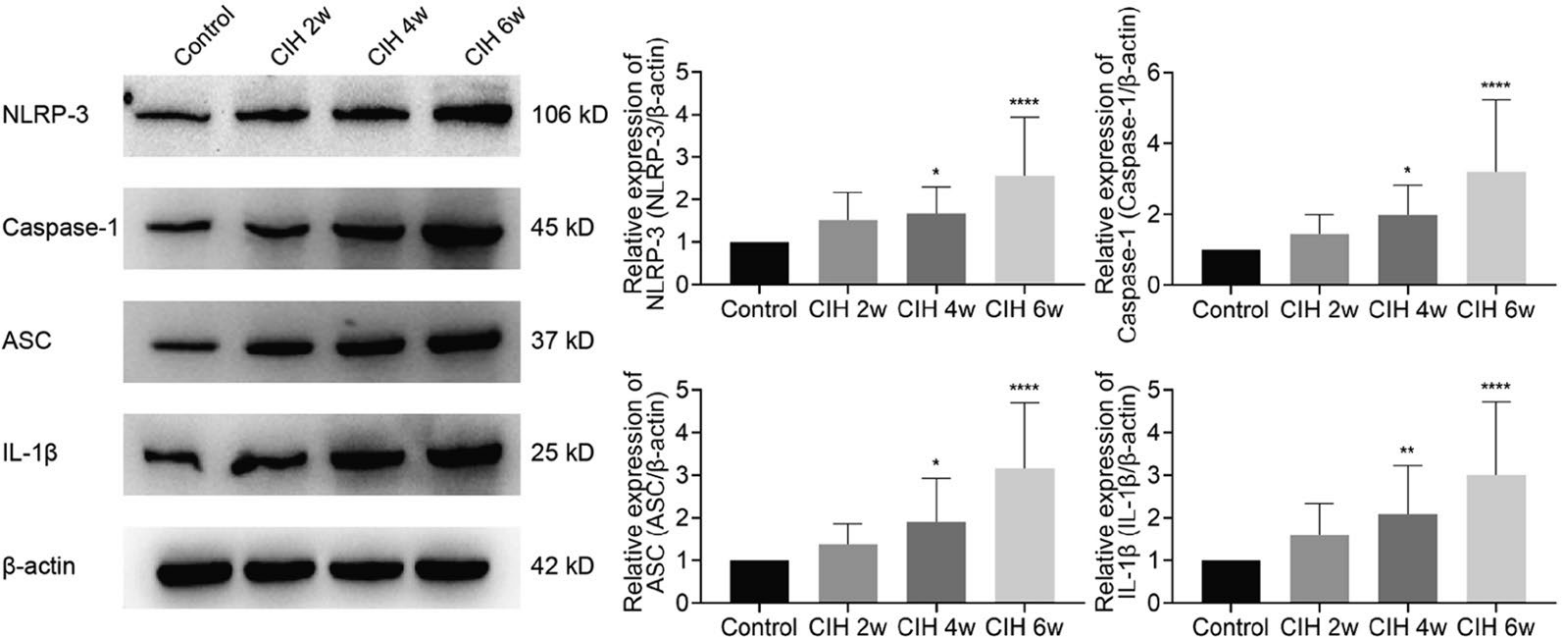
CIH induced increased NLRP3 inflammasome expression. Data are presented as the mean ± SD. ANOVA with Tukey’s post hoc test was used to compare the control, CIH 2w, CIH 4w, and CIH 6w groups. There were 5 rats per group, experiment was repeated 4 times. *P < 0.05, **P < 0.01, ****P < 0.0001

Western blotting showed that CIH could induce the activation of the NLRP3/Caspase-1/ASC/IL-1β pathway, while the expressions of NLRP3, Caspase-1, ASC, and IL-1β were significantly increased at CIH 4w (*P* < 0.05, *P* < 0.05, *P* < 0.05, *P* < 0.01) and CIH 6w (all *P* < 0.0001), as shown in Fig. 5. Based on this result, we speculated that the NLRP3/Casepae-1/ASC/IL-1β signaling pathway might have an important role in CIH-related brain injury.

### Inhibition of NLRP3 inflammasome ameliorates CIH-related brain injury, reduces astrocyte activation, and reverses the transformation from A2 to A1 of astrocytes

**Fig. 6:**
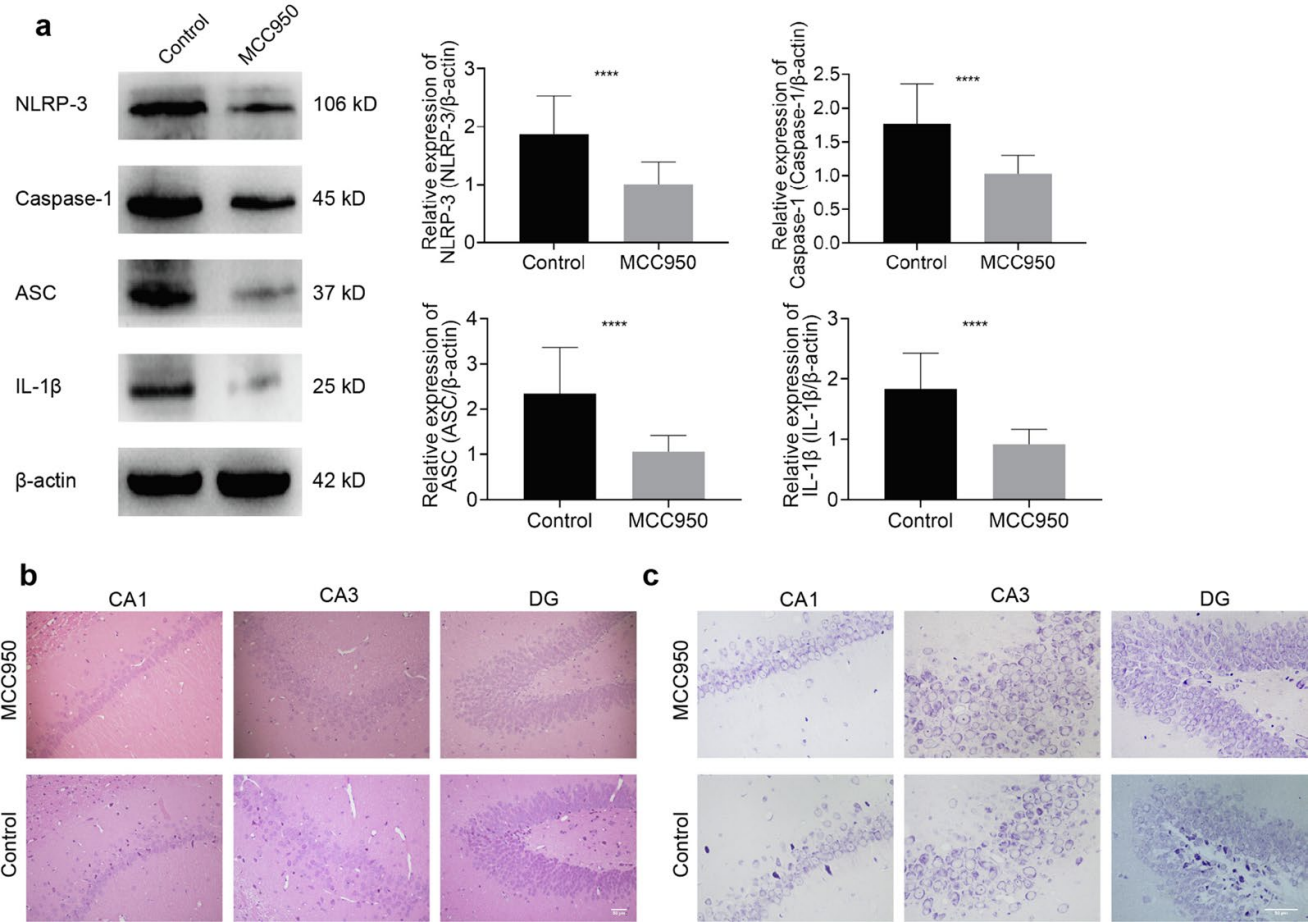
Inhibition of the NLRP3 inflammasome attenuated CIH-related brain injury. **a** The Western blotting for NLRP3, Caspase-1, ASC, and IL-1β in the hippocampus. **b** HE staining of different regions of the hippocampus. **c** Nissl staining of different hippocampal regions. Scale bar = 50 μm. Data are presented as the mean ± SD. There were 5 rats per group, experiment was repeated 4 times. For statistical analysis, an unpaired t test was used. ****P < 0.0001

In order to demonstrate that the neural damage caused by the inflammasome in the CIH model occurs through the regulation of astrocytes, we administered an NLRP3 inhibitor to conduct an intervention study. Western blotting showed that MCC950 could significantly inhibit the protein expression of NLRP3 inflammasome (all *P* < 0.0001, Fig. 6a), thus confirming that the drug intervention model was successfully constructed. Furthermore, histopathological staining showed that after MCC950 intervention, the number of hippocampal neurons increased. Moreover, they were neatly arranged (Fig. 6b) and accompanied by an increase in the number of Nissl bodies (Fig. 6c), which preliminarily suggested that inhibition of NLRP3 has a protective role in CIH-related brain injury.

**Fig. 7:**
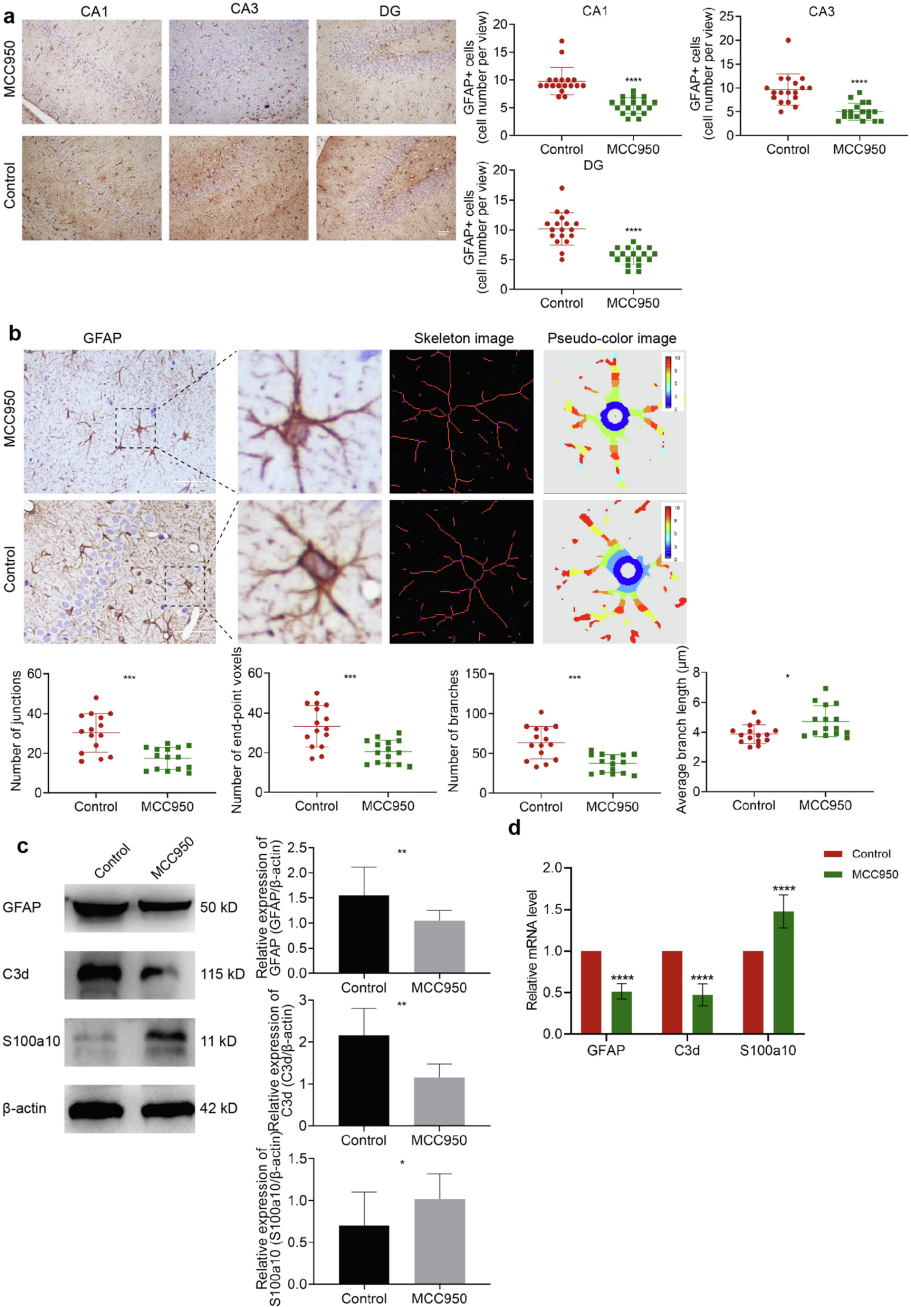
Inhibition of NLRP3 inflammasome reversed CIH-related A1/A2 astrocyte phenotypic transformation. **a** GFAP immunohistochemical staining and GFAP + cell count. **b** Sholl analysis of morphological changes of astrocytes. **c-d** Expression of GFAP, C3d, and s100a10 detected by Western blotting and RT-qPCR. Scale bar = 50 μm. Data are presented as the mean ± SD. For RT-qPCR experiments, each group consisted of 4 rats, and the experiment was repeated 3 times; for Western blotting, the experiment was repeated 4 times and there were 5 rats per group. For statistical analysis, an unpaired t-test was used. *P < 0.05, **P < 0.01, ***P < 0.001, ****P < 0.0001

Immunohistochemical results showed that after inhibiting the NLRP3 inflammasome, the number of astrocytes in each subregion of the hippocampus decreased (all *P* < 0.0001, Fig. 7a). Also, the Sholl analysis found that MCC950 drug intervention reduced the number of synaptic junctions (*P* < 0.001), synaptic end-point voxels (*P* < 0.001), and astrocyte synaptic branches (*P* < 0.001), while the average branch length increased (*P* < 0.05), as shown in Fig. 7b.

The results of Western blotting showed that compared with the control group, the expressions of GFAP and C3d proteins were significantly decreased (*P* < 0.01, *P* < 0.05), while the expression of S100a10 was increased (*P* < 0.05), as shown in Fig. 7c. The same trend was found at the mRNA level (Fig. 7d), with a decrease of GFAP (*P* < 0.0001) and C3d (*P* < 0.0001), and an increase of S100a10 (*P* < 0.0001).

## Discussion

OSA is a sleep disorder characterized by repetitive apnea, chronic hypoxia, oxygen desaturation, and hypercapnia. In the US, the prevalence of OSA is approximately 26.6% in men and 8.7% in women aged 30—49 years old and about 43.2% in men and 27.8% in women aged 50—70 years old [21]. CIH is the unique pathological mechanism of OSA. The repeated hypoxia and reoxygenation in the whole pathological process have a pathophysiological mechanism similar to that of ischemia–reperfusion injury, resulting in persistent oxidative stress and cascade inflammatory response in the body. Neurocognitive dysfunction is an essential complication of OSA. Using functional imaging techniques, scholars have found that OSA patients with neurocognitive deficits often have extensive structural changes in brain regions [22]. The hippocampus is the most sensitive area of brain tissue hypoxia, whose structural subregions CA1 and DG regions of the dentate gyrus are considered as most closely related to cognitive function. Macey et al*.* [23] found that in newly diagnosed OSA patients who did not receive treatment, increases and decreases in volume occurred in the early and emerging regions of the hippocampus.

In this study, the hippocampus was positioned as the most critical observation area for CIH-related brain injury. HE and Nissl staining showed that under CIH, the arrangement of hippocampal neurons was disordered. Also, there was some nuclear pyknosis and fragmentation, while the number of Nissl bodies was reduced. At the same time, the results of animal water maze experiments indicated that CIH could lead to the decline of spatial learning ability, which is consistent with previous literature results [24, 25].

Astrocytes exert multiple important roles in regulating homeostasis, increasing synaptic plasticity, and providing neuroprotection. At the same time, astrocytes can participate in the inflammatory response, which has a key role in the progression of psychiatric and neurodegenerative diseases [26, 27]. After stimulation, resting astrocytes transform into reactive astrocytes. Recent studies suggested dividing reactive astrocytes into two types, i.e., A1 neurotoxic astrocytes marked by C3d can highly upregulate many classical complement cascade genes and induce neural damage, and A2-type astrocytes labeled by S100a10, known as neuroprotective glial cells, can release many neurotrophic factors and regulate brain homeostasis [28]. C3d, a fragment of complement component C3, has an essential role in the induction and regulation of immune responses [29]. S100a10, a uniquely controversial member of the S100 EF-hand protein family, regulates innate inflammatory and immune responses [30]. Activated astrocytes are thought to be involved in various neurologically damaging diseases. Our findings revealed that CIH activates astrocytes and simultaneously leads to an increase in A1-type astrocytes. A1-type astrocytes are thought to have lost normal astrocyte function but acquired new toxic functions, which were expressed in various neurodegenerative diseases, and induced neurodegenerative disease progression [31]. Previous studies have found that A1 reactive astrocytes secrete inflammatory factors such as IL-1β, IL-6, IFN-γ and TNF-α as well as classical complement cascade genes and some unknown toxicity factors, thus inducing cognition dysfunction [32]. Therefore, combined with previous studies and our findings, it can be speculated that astrocytes may be important target cells in CIH-related brain injury, where the increased expression of A1 neurotoxic astrocytes may be an important pathological mechanism.

Neuroinflammation is initiated with an aberrant innate immune response in the CNS and is involved in many neurological diseases. Inflammasomes are intracellular multiprotein complexes that can be used as platforms to induce the maturation and secretion of proinflammatory cytokines and pyroptosis, thus having a pivotal role in neuroinflammation. Among inflammasomes, the NLRP3 inflammasome is reported to sense a wide range of stimuli and be involved in various diseases [33]. Liu et al*.* [34] found that the degree of neuroinflammation and synaptic damage in the hippocampus in depressed rats was related to the activation of the NLRP3 inflammasome and inhibition of the NLRP3 inflammasome could reverse the related damage. It was also reported that inhibition of NLRP3 inflammasome could improve brain tissue damage and maintain blood–brain barrier integrity in Parkinson’s [35] and ischemia–reperfusion brain injury [36]. Consistent with the above studies, our results revealed that the NLRP3 inflammasome was over-expressed in CIH-related brain injury. After inhibiting NLRP3 with MCC950 [37], the pathological damage of brain tissue was significantly alleviated. These results suggested that CIH-related brain injury may be attributed to the activation of the NLRP3 inflammasome. However, the exact mechanism of how the NLRP3 inflammasome induces neuronal damage remains unclear.

Early studies suggested that the NLRP3 inflammasome is only expressed in the central nervous system microglia and has a role in regulating neuroinflammation. Current studies have shown that astrocytes can assemble different inflammasomes and participate in various neurological diseases [38, 39]. In the present study, we further analyzed the changes of astrocytes before and after NLRP3 inhibition in the CIH model, finding that inhibition of NLRP3 could alleviate CIH-induced astrocyte activation and increase the expression of A2-type astrocytes. These results suggest that NLRP3 regulates CIH-related brain injury by activating astrocytes and inducing increased A1-type astrocyte expression.

Our findings suggest that the NLRP3 inflammasome has an important role in CIH-related brain injury, and inhibition of the NLRP3 inflammasome may be a therapeutic target for alleviating and preventing CIH-related brain injury. At the same time, from the perspective of neuroinflammation, we used astrocytes as target cells and found phenotypic changes in A1/A2 astrocytes in CIH-induced brain injury. Also, our findings provide a new perspective for scholars to explore the research of CIH-related brain injury. However, the present study was only limited to astrocytes. In future studies, scholars should focus on the neurovascular unit and explore the interaction between glial cells, neurons, and blood vessels.

## Conclusions

Our results unequivocally confirm that CIH can activate the NLRP3/Caspase-1/ASC/IL-1β pathway, reduce the number of A2 astrocytes and increase the number of A1 astrocytes, thus resulting in brain tissue damage. Furthermore, inhibition of NLRP3 inflammasome modulates phenotypic transformation of A1/A2 astrocytes, thereby reversing CIH-related brain injury. Taken together, these results suggest that NLRP3 inflammasome regulation of A1/A2 astrocyte phenotype transformation is an important mechanism of CIH-related brain injury.

## Supplementary Information

**Additional file 1**: **Figure S1**. Research Supplementary Materials. a Process diagram of rat CIH model b Weight Trend Chart of Rat. ****P<0.0001.

**Additional file 2**: **Figure S2**. Raw western blots of GFAP (a), C3d (b), S100a10(c) and β-actin (d) in Figure 4b 12% Gel, 0.45 µm PVDF membrane.

**Additional file 3**: **Figure S3**. Raw western bloats of NLRP3 (a), Capase-1 (b), ASC(c), IL-1β and β-actin (e) in Figure 5 The NLRP3, Capase-1, β-actin used 10% Gel; ASC, IL-1β used 12% Gel; all used 0.45 µm PVDF membrane.

**Additional file 4**: **Figure S4**. Raw western bloats of NLRP3 (a), Capase-1 (b), ASC(c), IL-1β and β-actin (e) in Figure 6a. The NLRP3, Capase-1, β-actin used 10% Gel; ASC, IL-1β used 12% Gel; all used 0.45 µm PVDF membrane.

**Additional file 5: Figure S5.** Raw western blots of GFAP(a), C3d (b), S100a10(c) and β-actin (d) in Figure 7c 12%Gel, 0.45 µm PVDF membrane.

**Additional file 6: Table S1.** The primer sequences uesd in RT-qPCR.

**Additional file 7.** Supplementary materials and methods.

## Data Availability

The datasets generated and/or analyzed during the current study are not publicly available due to ongoing research projects but are available from the corresponding author on reasonable request.

## Abbreviations

OSA: Obstructive sleep apnea
CIH: Chronic intermittent hypoxia
NLRP3: Nucleotide oligomerization domain (NOD)-like receptor protein 3
ASC: Apoptosis-associated speck-like protein
GFAP: Recombinant glial fibrillary acidic protein
RT-qPCR: Reverse transcription‑quantitative PCR
CNS: Central nervous system
SD: Sprague–Dawley
DMSO: Dimethyl sulfoxide
HE: Hematoxylin eosin
