# Supplementary figures and images for: NLRP3 inflammasome regulates astrocyte transformation in brain injury induced by chronic intermittent hypoxia
Source: BMC Neurosci. 2022 Nov 27;23:70. doi: 10.1186/s12868-022-00756-2 (PMC9703760; doi:10.1186/s12868-022-00756-2)

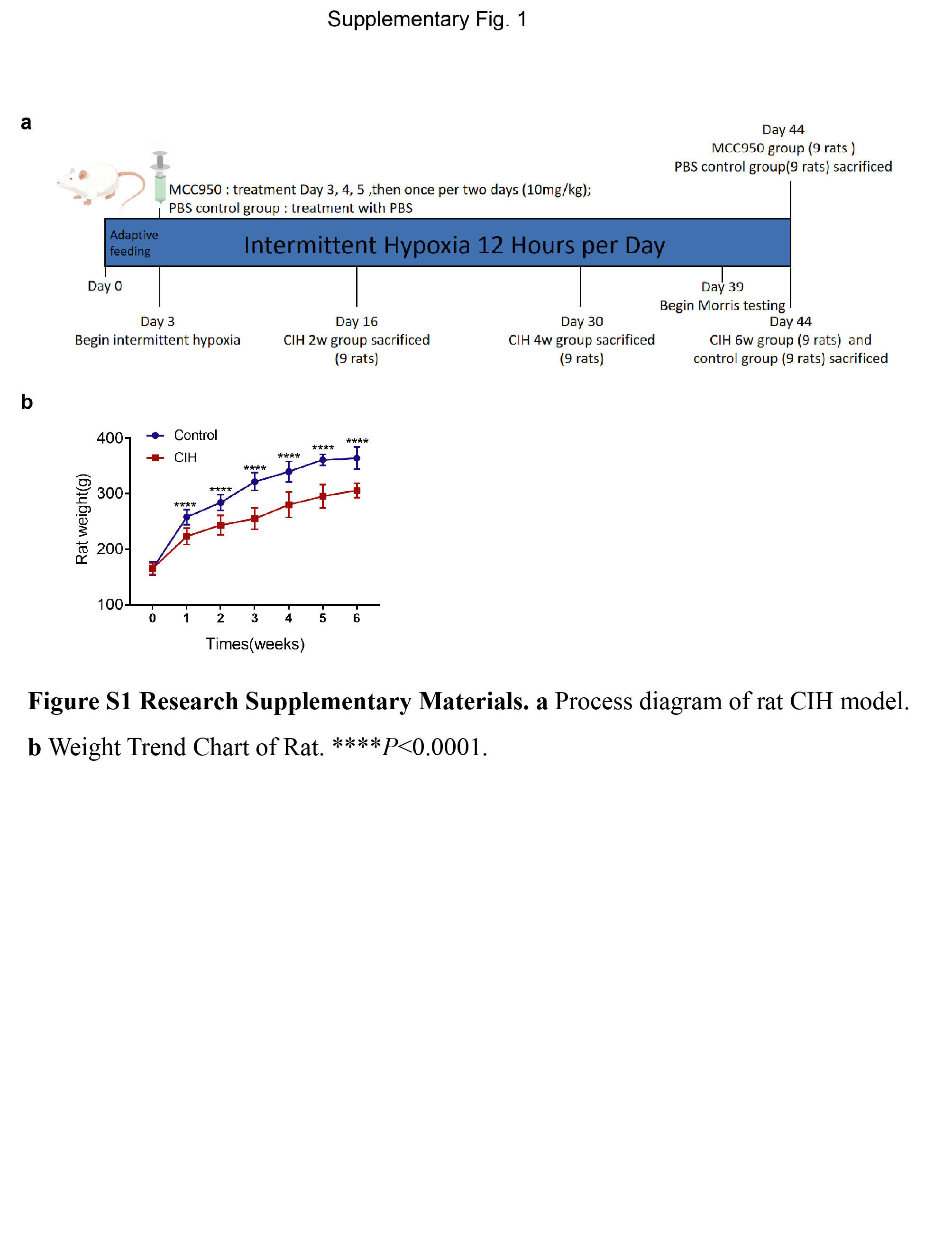

Supplement: Supplementary file 1 — Additional file 1: Figure S1. Research Supplementary Materials. a Process diagram of rat CIH model b Weight Trend Chart of Rat. ****P<0.0001. [file 12868_2022_756_MOESM1_ESM.jpg]

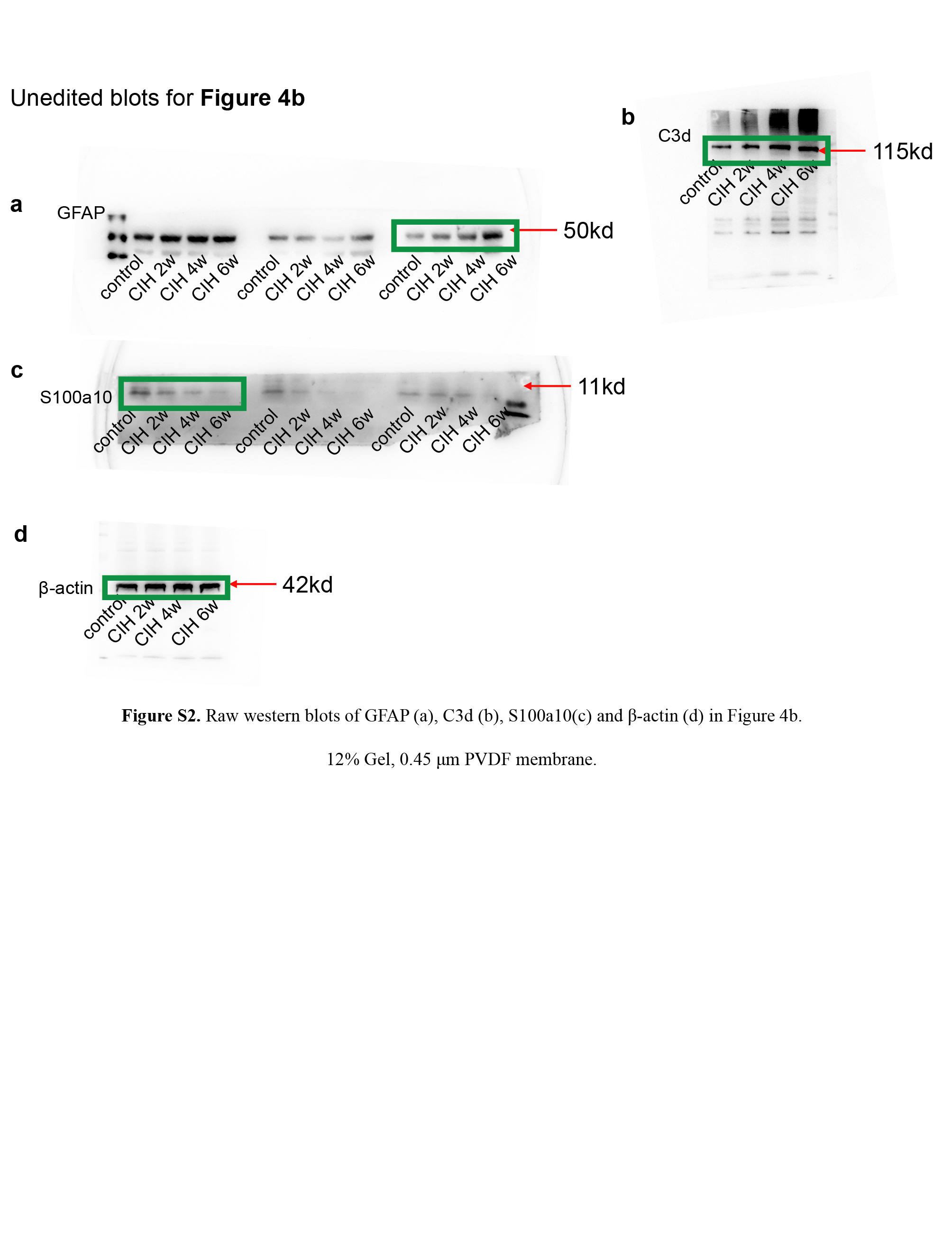

Supplement: Supplementary file 2 — Additional file 2: Figure S2. Raw western blots of GFAP (a), C3d (b), S100a10(c) and β-actin (d) in Figure 4b 12% Gel, 0.45 µm PVDF membrane. [file 12868_2022_756_MOESM2_ESM.jpg]

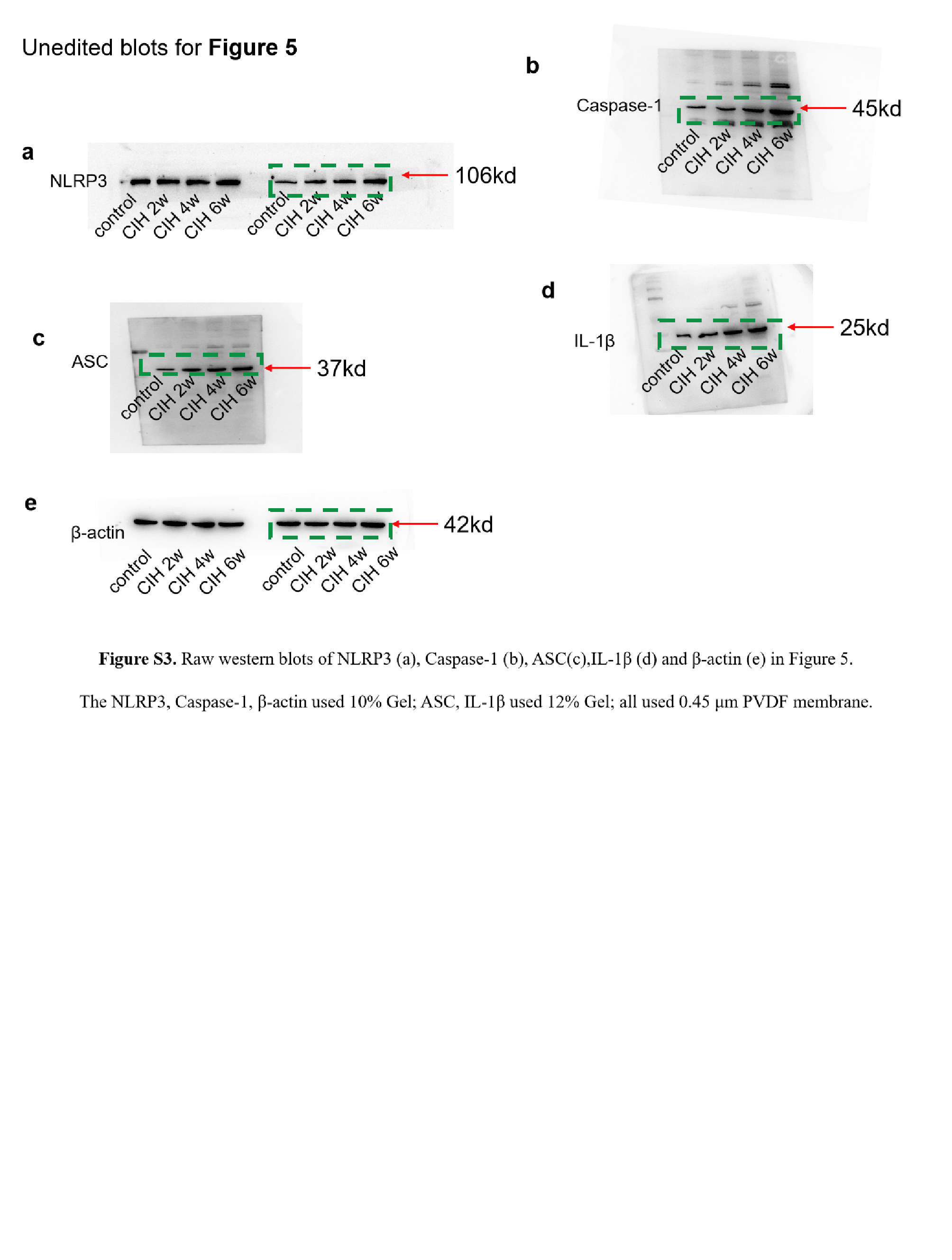

Supplement: Supplementary file 3 — Additional file 3: Figure S3. Raw western bloats of NLRP3 (a), Capase-1 (b), ASC(c), IL-1β and β-actin (e) in Figure 5 The NLRP3, Capase-1, β-actin used 10% Gel; ASC, IL-1β used 12% Gel; all used 0.45 µm PVDF membrane. [file 12868_2022_756_MOESM3_ESM.jpg]

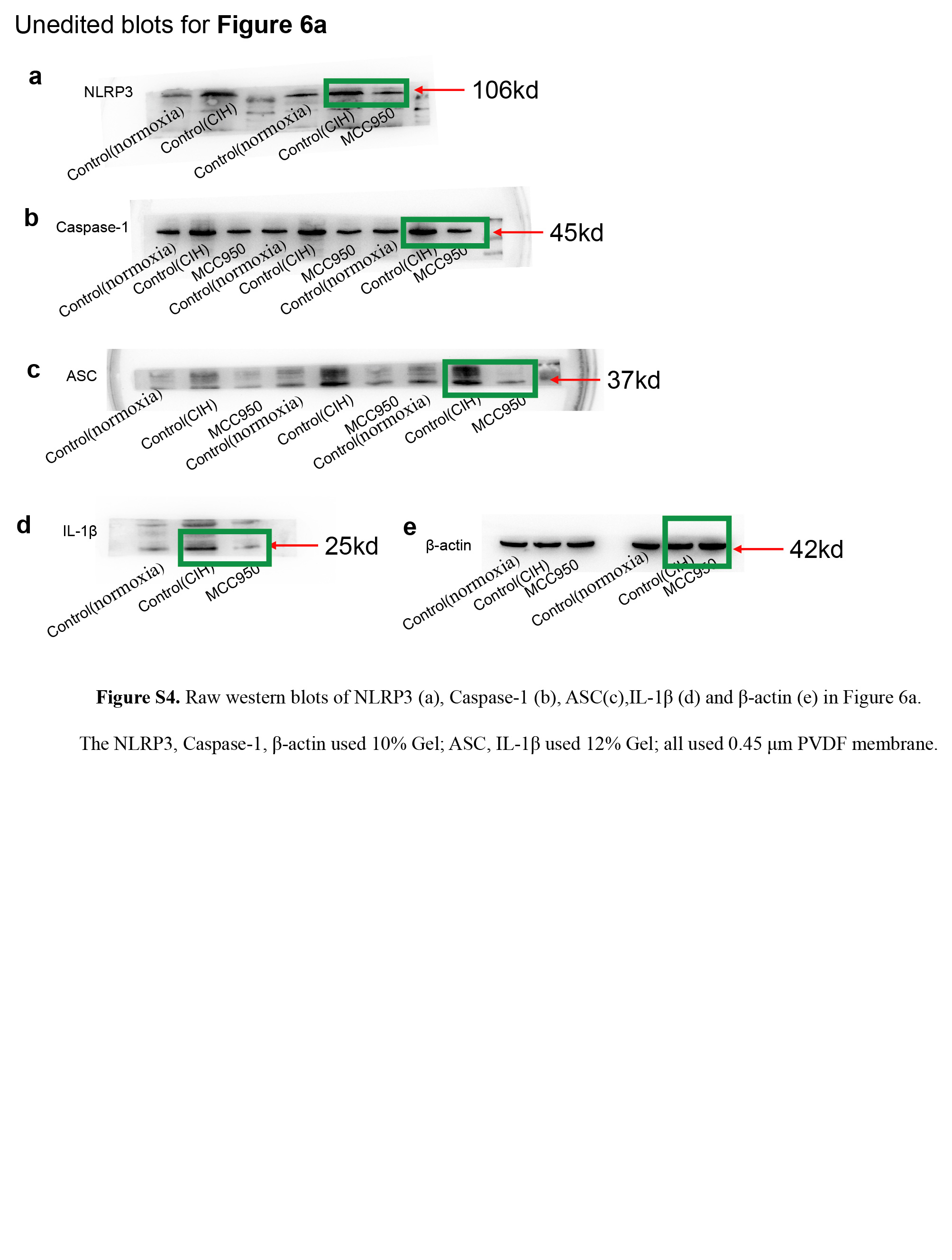

Supplement: Supplementary file 4 — Additional file 4: Figure S4. Raw western bloats of NLRP3 (a), Capase-1 (b), ASC(c), IL-1β and β-actin (e) in Figure 6a. The NLRP3, Capase-1, β-actin used 10% Gel; ASC, IL-1β used 12% Gel; all used 0.45 µm PVDF membrane. [file 12868_2022_756_MOESM4_ESM.jpg]

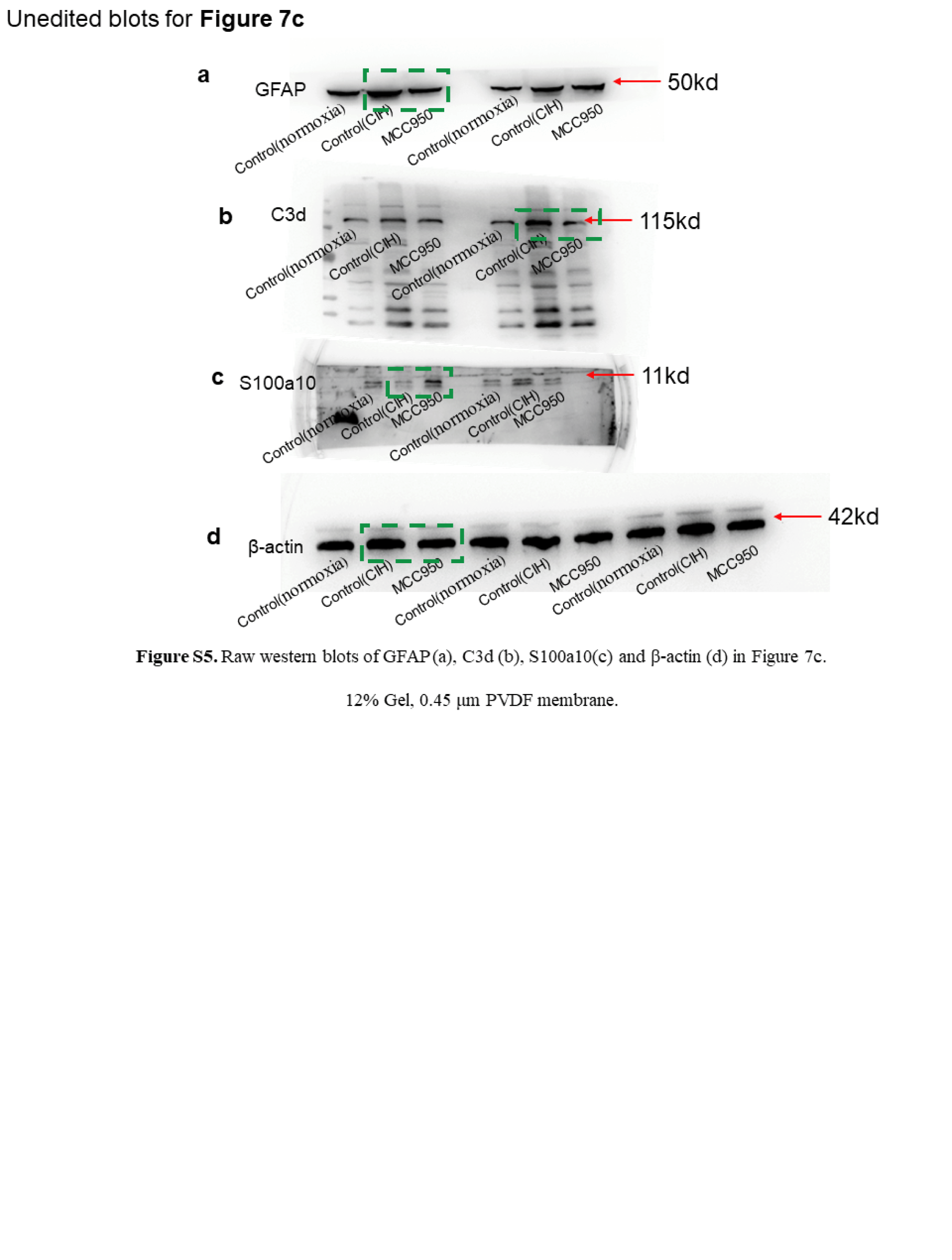

Supplement: Supplementary file 5 — Additional file 5: Figure S5. Raw western blots of GFAP(a), C3d (b), S100a10(c) and β-actin (d) in Figure 7c 12%Gel, 0.45 µm PVDF membrane. [file 12868_2022_756_MOESM5_ESM.tif]
