# Supplementary material for: NLRP3 inflammasome regulates astrocyte transformation in brain injury induced by chronic intermittent hypoxia
Source: BMC Neurosci. 2022 Nov 27;23:70. doi: 10.1186/s12868-022-00756-2 (PMC9703760; doi:10.1186/s12868-022-00756-2)
Supplement: Supplementary file 7 — Additional file 7. Supplementary materials and methods. [file 12868_2022_756_MOESM7_ESM.pdf]

Materials and methods

Western blotting

Rats were anesthetized with an intraperitoneal injection of 1% (w/v) pentobarbital sodium (35 mg/kg), and the bilateral hippocampus was immediately removed and stored in liquid nitrogen until further processing. The protein was extracted under ultrasound using Radio-Immunoprecipitation Assay lysis buffer (RIPA, Sigma, USA), and the protein concentration was determined by the bicinchoninic acid (BCA, Beyotime, China) method. Protein samples were separated by 10% and 12% sodium dodecyl sulfate-polyacrylamide gel electrophoresis (SDS-PAGE gel electrophoresis), after which the proteins were transferred to 0.45 um polyvinylidene fluoride (PVDF) membranes (Millipore Corp, Billerica, MA, USA). Membranes were blocked with 10% nonfat dry milk in TBST buffer for 1 h at room temperature, followed by blocking with primary antibody overnight at 4°C. The membrane was then washed with TBST and combined with the secondary antibody for 1 h at room temperature with subsequent washing with TBST. Then, the membranes were visualized using a ChemiDoc MP System (Bio-Rad protein assay, Bio-Rad, Italy) with ECL substrate (Omni-ECL™Femto Light Chemiluminescence Kit, Epizyme Biomedical Technology, China). For some proteins, there are some non-specific bands after ECL luminescence, we strictly according to the protein molecular weight to select the corresponding position of the bands for analysis,

and show in the manuscript. And some of the more difficult to expose strips, appropriately extend the luminous time. β-actin was chosen as an internal control to ensure equivalent amounts of protein, and densitometric quantification of the bands was performed using Image J software. Primary antibody information were: NLPR3 (cat. no. WL02635, 1:1000, Wanleibio, China), caspase-1 (cat. no. sc-56036, 1:1000, Santa Cruz, USA), ASC (cat. no. sc-514414, 1:500, Santa Cruz, USA), IL-1β (cat. no. sc-12742, 1:500, Santa Cruz, USA), GFAP (cat. no. BM0055, 1:1000, Boster biological technology, China), S100a10 (cat. no. 11250-1-AP, 1:1000, Proteintech, China), C3d (cat. no. AF2655, 1:1000, RD system, USA), and β-actin (cat. no. BM3873, 1:10,000, Boster biological technology, China).

Hematoxylin eosin stain

Rats were anesthetized with pentobarbital sodium and transcardially perfused with 250 mL normal saline and 250 mL 4% paraformaldehyde (PFA) in 0.1 mol/L phosphate buffer. Next, their brains were harvested, fixed with PFA, embedded in paraffin, and cut into sections (5 μm) with a rotary microtome. The prepared rat brain tissue paraffin sections were selected, dewaxed, hydrated, stained with hematoxylin-eosin, further dehydrated, transparent, mounted, and observed under a light microscope. Under a 20× optical microscope, two fields of view were randomly selected for each film in different subregions of hippocampal CA1, CA3, and DG.

Immunohistochemical staining

Sections were routinely dewaxed and washed, and antigen retrieval was performed by microwave (sodium citrate buffer, medium-high heat for 8 min, stop for 7 min, medium-low heat for 8 min), and naturally cooled to room temperature. They were washed with PBS three times, then blocked in endogenous peroxidase with 3% H_2O_2 for 20 min at room temperature, washed three times with PBS, and blocked non-specific protein binding with normal goat serum at 37°C for 30 min. Samples were subsequently incubated with mouse anti-GFAP polyclonal antibody (cat. no. BM0055, 1:200, Boster biological technology, China) at 4°Covernight. Sections were then incubated with goat anti-mouse IgG biotin for 20 minutes and washed three times with PBS. Consequently, sections were incubated with streptavidin-horseradish peroxidase for 20 minutes at room temperature. Diaminobenzidine was used as a developer, and hematoxylin was used as a counterstain. Finally, the sections were sequentially dehydrated and transparent, covered with a coverslip, and observed under a light microscope. For each specimen, 5-6 different fields of view were randomly selected according to different hippocampal regions (CA1, CA3, DG) under a 40× microscope to count the number of $GFAP^+$ cells. Under a 40× microscope, 5 morphologically intact astrocytes were randomly selected from each specimen for Sholl analysis.
